# Supplementary material for: The psychological status of patients with delayed intravitreal injection for treatment of diabetic macular edema due to the COVID–19 pandemic
Source: PLoS One. 2023 Aug 25;18(8):e0290260. doi: 10.1371/journal.pone.0290260 (PMC10456183; doi:10.1371/journal.pone.0290260)
Supplement: S1 File — (PDF) [file pone.0290260.s002.pdf]

ResearchGate

See discussions, stats, and author profiles for this publication at: https://www.researchgate.net/publication/342569857

Mental Health State of Low Vision Patients using the Hospital Anxiety and Depression Scale and The Depression, Anxiety and Stress Scale

Article · June 2020

DOI: 10.17576/MH.2020.1501.18

CITATIONS
0

READS
356

1 author:

Mohd Harimi Abd Rahman
Universiti Kebangsaan Malaysia

18 PUBLICATIONS **64** CITATIONS

SEE PROFILE

Some of the authors of this publication are also working on these related projects:

Project

UNRAVELLING THE ROLE OF AUDITORY AND VISUAL PROCESSING AS POTENTIAL FUNCTIONAL BIOMARKERS OF COGNITIVE AGEING View project

All content following this page was uploaded by Mohd Harimi Abd Rahman on 02 July 2020.

The user has requested enhancement of the downloaded file.

https://doi.org/10.17576/MH.2020.1501.18

ORIGINAL ARTICLE

Mental Health State of Low Vision Patients using the Hospital Anxiety and Depression Scale and The Depression, Anxiety and Stress Scale

MOHD HARIMI AR[1], NADHRAH AB[1], NUR HIDAYAH Y[1], ZAINORA M[1], JEMAIMA CH[2], MUSHAWIAHTI M[2]

[1]Optometry and Visual Science Programme, Faculty of Health Sciences, Universiti Kebangsaan Malaysia Kampus Kuala Lumpur (UKMKL), Jalan Raja Muda Abdul Aziz, 50300 Kuala Lumpur, Malaysia.
[2]Department of Ophthalmology, Faculty of Medicine, Universiti Kebangsaan Malaysia Medical Centre, Jalan Yaacob Latif, Bandar Tun Razak, 56000 Cheras, Kuala Lumpur, Malaysia.

ABSTRAK

Penilaian status kesihatan mental dalam kalangan pesakit penglihatan terhad (LV) bukan merupakan rutin pemeriksaan. Kajian ini dijalankan untuk mengenal pasti status kesihatan mental dalam kalangan pesakit LV menggunakan soal selidik "Depression Anxiety Stress Scale (DASS-21) and Hospital Anxiety and Depression (HAD) Scale". Seramai 100 pesakit LV telah dipilih secara rawak. Pemeriksaan piawai LV dan penilaian kesihatan mental menggunakan soal selidik DASS-21 dan HAD scale telah dijalankan. Purata umur subjek adalah 62.77±9.15 tahun. Subjek terdiri daripada 63% Melayu, diikuti 26% Cina dan 11% India. Purata tahap penglihatan subjek adalah 0.45±0.24 LogMAR. Keputusan kajian menunjukkan min skor kemurungan dan kerisauan bagi soal selidik HAD Scale adalah 3.11±3.35 dan 2.85±3.21. Min skor kemurungan, kegelisahan dan tekanan bagi soal selidik DASS adalah 4.83±6.90, 3.58±3.79 dan 6.18±6.92. Ini menunjukkan min skor bagi kedua-dua soal selidik adalah dalam julat normal mengikut klasikasi tahap keterukan HAD dan DASS. Soal selidik HAD Scale menunjukkan 12% daripada subjek mengalami kemurungan dan 8% mengalami kegelisahan, manakala soal selidik DASS menunjukkan 7% mengalami kemurungan, 17% mengalami kegelisahan dan 24% mengalami tekanan. Ini menggambarkan bahawa kesihatan mental pesakit LV boleh terjejas disebabkan oleh gangguan penglihatan. Kesimpulannya, status kesihatan mental pesakit LV boleh ditentukan secara objektif menggunakan soal selidik DASS-21 dan HAD Scale. Penentuan status ini juga memastikan rawatan dan rehabilitasi yang lebih optimum dapat diberikan kepada pesakit LV.

Address for correspondence and reprint requests: Mohd Harimi Abd Rahman. Optometry and Visual Science Programme, Center for Rehabilitation and Special Needs, Faculty of Health Sciences, Universiti Kebangsaan Malaysia Kampus Kuala Lumpur, Jalan Raja Muda Abdul Aziz, 50300 Kuala Lumpur, Malaysia. Tel: +603-9289 7157 Email: harimirahman@gmail.com

Kata kunci: *DASS, gangguan penglihatan, kesihatan mental, penglihatan terhad, skala HAD*

ABSTRACT

Mental health status of low vision (LV) patients is not a routine evaluation. This study was carried out to determine the mental health status among LV patient using Depression Anxiety Stress Scale (DASS-21) and Hospital Anxiety and Depression (HAD) Scale questionnaires. A total of 100 LV patients were recruited in this cross-sectional study. Standard LV assessment and mental health screening using DASS-21 and HAD Scale questionnaires were administered. The mean age of the subjects was 62.77±9.15 years old. There were 63% Malays, followed by 26% Chinese and 11% Indians. The mean visual acuity of the subjects was 0.45±0.24 LogMAR. Results showed the mean score for depression and anxiety for HAD scale questionnaire were 3.11±3.35 and 2.85±3.21, respectively. The mean score for depression, anxiety and stress for DASS questionnaire were 4.83±6.90, 3.58±3.79 and 6.18±6.92, respectively. The mean scores fell into the normal range classification of severity on both HAD scale and DASS. The HAD Scale showed that 12% of the subjects experienced depression and 8% experienced anxiety while the DASS showed that 7% had depression, 17% had anxiety and 24% experienced stress. These findings illustrate that the mental health of the LV patients can be affected due to visual impairment. In conclusion, the mental health status of LV patients can be screened objectively using DASS-21 and HAD Scale questionnaire. Understanding the mental health status of LV patients will enable us to provide better LV assessment and rehabilitation.

Keywords: DASS, HAD scale, low vision, mental health, visual impairment

INTRODUCTION

According to World Health Organization (WHO), low vision (LV) was defined as visual acuity (VA) of worse than 6/12 but equal to or greater than 6/60, or a corresponding visual field loss to less than 20 degrees in the better eye with the best correction (WHO 2008). Global Vision Database estimated that in the year 2010, there were 32.4 million people with blindness and 191 million with LV (Stevens et al. 2013) but has increased to 36 million people with blindness and 217 million people with LV in the year 2015 (Bourne et al. 2017). Globally, the major causes of LV were uncorrected refractive errors (43%), cataract (33%) and glaucoma (2%) (WHO 2012). Other causes include diabetic retinopathy, age-related macular degeneration (ARMD), trachoma and corneal opacities each accounted for about 1% (WHO 2012). The increasing prevalence of LV warranted studies

on this populations as LV has been associated with negative outcomes such as mental health problems and reduced quality of life (QoL) (Rishi et al. 2017; Omar et al. 2014; Dibajnia et al. 2013; Noran et al. 2009).

Depression and anxiety are part of the elements of mental health status which are considered serious medical conditions as they may exacerbate disability caused by visual impairment, increase vulnerability for health deterioration and reduce QoL, which lead to poor LV rehabilitation outcomes (Jones et al. 2009; Breslin et al. 2006; Strine et al. 2004). LV is associated with increased depressive and anxiety symptoms as vision loss has a direct impact on physical disability and restrictions in daily living activities (Evans et al. 2007; Augustin et al. 2007; Horowitz et al. 2005). People with LV who suffered from mental-health problems were often not identified and therefore, not referred for mental health services. The main reasons mental health interventions needs were unmet were lack of awareness and self-reliance (van der Aa et al. 2015). The status of the mental health should be determined during the LV assessment as it is an imperative step to optimise the management of LV.

There are some screening tools that can objectively assess mental health status such as Depression Anxiety Stress Scale (DASS), Hospital Anxiety and Depression (HAD) Scale, Geriatric Depression Scale (GDS) and General Health Questionnaire (GHQ-28) (Goldberg et al. 1997; Lovibond & Lovibond 1995; Zigmond & Snaith 1983; Yesavage et al. 1982). DASS is a simple and appropriate screening tool to be directed to general population (Ramli et al. 2012; Lovibond & Lovibond 1995). Previous studies found that the Malay version of DASS-21 had good psychometric properties among clinical and non-clinical populations and good validity for most of its elements (Ramli et al. 2011; Ramli et al. 2007). The HAD Scale was found to be reliable in providing a brief assessment on severity of depression and anxiety in the hospital setting (Rishi et al. 2017). All 14 items of HAD Scale questionnaire has a good degree of internal consistency among the Malaysian population (Ramli et al. 2011). Both DASS and HAD Scale questionnaires have been used as screening tools in studies on mental health among LV patients (Rishi et al. 2017; Omar et al. 2014; Kempen et al. 2012). However, there is still no study on the comparison between DASS and HAD Scale among LV population.

Currently, screening of mental health status is not routine in history taking and there is no standard screening questionnaire to objectively measure mental health status in LV assessment (Omar et al. 2014). Therefore, mental health status often left undetected and untreated among visually impaired patients (Yahya & Othman 2015; Rovner 2002). Thus, this study aims to determine the mental health status among LV patients using DASS and HAD Scale questionnaires. Thus, a suitable screening tool for mental health in LV assessment can be identified so that multidisciplinary approach of patient's management is achieved.

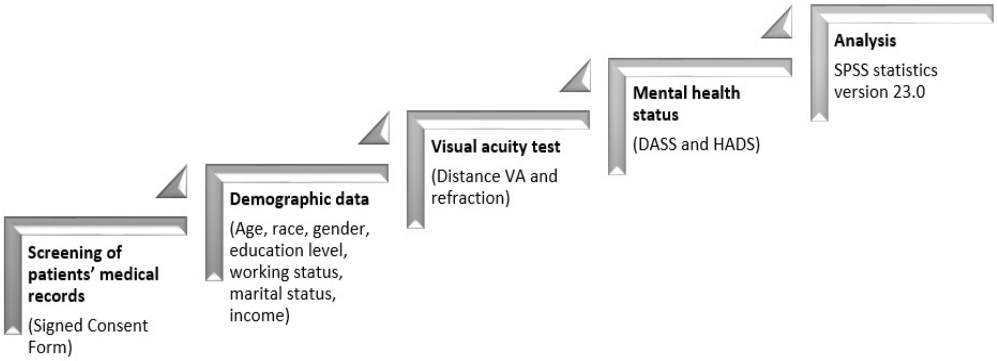

Figure 1: Flow chart of research procedure

MATERIALS AND METHODS

A total of 100 LV subjects aged 40 years and above were recruited from the Ophthalmology Clinic at Hospital Canselor Tuanku Muhriz (HCTM) in this cross-sectional study. The sampling method used is purposive sampling. Screening of the medical records of the subjects was carried out in order to choose the suitable subjects that fit the criteria. Subjects who fulfilled the criteria were invited to the ophthalmology clinic at HCTM and were given an explanation about the study through the Patient Information Sheet. There were 50 subjects diagnosed with diabetic retinopathy and 50 with glaucoma. The LV subjects with moderate visual impairment which visual acuity (VA) worse than 6/18 but better than or equal to 6/60 or mean deviation of Humphrey Visual Field (HVF) between -6 and -20 in the better eye with best correction were chosen for this study. Patients must be able to understand and communicate in Malay language and attain a score of 17 and above in Malay-Mini Mental State Examination (M-MMSE). Patients who had received any vision rehabilitation interventions and have chronic diseases such as stroke, chronic kidney disease, advanced lung disease and hypertension were excluded from the study. This study adhered to the Declaration of Helsinki and ethical approval was obtained from the Research Ethics Committee, Universiti Kebangsaan Malaysia (UKM) with research code UKM PPI/111/8/JEP-2018-433. Informed written consent was obtained from all patients.

The flow chart of the procedures is presented in Figure 1. Screening of subjects' medical records was carried out to choose subjects that fit the criteria. The demographic data on gender, age, race and medical history were collected by face-to-face interviews. The best distance VA was tested using the Early Treatment Diabetic Retinopathy Study (ETDRS) chart at 3 m and the visual field was tested using Humphrey Field Analyzer (HFA) by Carl Zeiss Meditec. The DASS and HAD Scale questionnaires were then administered to the subjects by face-to-face interviews.

The DASS-21 is a short version of

Table 1: Number of patients according to number of mental health problems using DASS and HAD Scale

Questionnaire	Number of mental health problem	Number of subjects, % (n=100)
DASS	None	74
	One	13
	Two	7
	Three	6
HAD Scale	None	84
	One	12
	Two	4

DASS-42 self-reported questionnaire used for screening of mental health status. It is designed to assess three emotional states of depression, anxiety and stress. Each of the three scales consists of seven items. Frequency or severity ratings were made on a series of 4-point scales (0=did not apply to me at all, 1=applied to me to some degree, or some of the time, 2=applied to me to considerable degree or a good part of time, 3=applied to me very much or most of the time). The scores were classified into normal, mild, moderate, severe and extremely severe based on a study by Omar et al. (2014).

The HAD Scale is a 14-item questionnaire designed to provide a brief assessment of anxiety and depression in out-patient populations (Zigmond & Snaith 1983). Each subscale includes seven items which were rated with a score from 0 to 3, with three denoting highest anxiety or depression level. The HAD Scale questionnaire was administered by the examiner to the patients in a face-to-face interview. The scores of depression and anxiety were calculated by summing the rating for each scale. A minimum cut off points of eight out of a possible 21 denotes considerable symptoms of anxiety and depression (Ng 2014).

The statistical analyses were performed using SPSS statistics version 23.0. The descriptive statistics were used to present the demographic data and the mental health scores of DASS and HAD Scale questionnaires. Mann-Whitney U was used for comparison mental health status between subjects with diabetic retinopathy and glaucoma.

RESULTS

There were 100 subjects enrolled in this study. The age range was 40 to 82 years old with a mean age of 62.77±9.15 years old. There were 45% female and 55% male patients. The demographic data show that majority of the subjects were Malay (63%), followed by Chinese (26%) and Indians (11%). The mean visual acuity of the subjects was 0.45±0.24 LogMAR.

Mental Health Status among Low Vision Patients

Table 2: Number of patients based on subscales of DASS

Severity level of DASS	Number of patients, % (n=100)		
	Depression	Anxiety	Stress
Normal	93	83	76
Mild	2	5	7
Moderate	3	11	11
Severe	0	1	4
Very severe	2	0	2

Based on the DASS-21 questionnaire, the mean scores for depression, anxiety and stress were 4.83±6.90, 3.58±3.79 and 6.18±6.92, respectively. The results showed that 26% of the subjects suffered at least one of mental health problem (Table 1).

Based on the three subscales of the DASS questionnaire, there were 7% subjects experienced depression, 17% experienced anxiety and 24% experienced stress. Table 2 shows the number of the subjects with different severity of depression, anxiety and stress. The median scores DASS for depression, anxiety and stress among LV patients with were 2.00±6.00 (range 0 to 36), 2.00±4.00 (range 0 to 18) and 4.00±6.00 (range 0 to 32), respectively. Friedman two way indicated that the score of DASS varied significantly across the three domains, χ^2=30.32, df=2, p=0.000.

Based on the HAD Scale questionnaire, the mean score for depression and anxiety subscales were 3.11±3.35 and 2.85±3.21, respectively. The results showed that 12% of the subjects experienced depression and 8% of the subjects experienced anxiety. There were 16% of the subjects experienced depression and/or anxiety as presented in Table 1. There were four subjects with both depression and anxiety. The median scores HAD scale for depression and anxiety among LV patients were 2.00±4.00 (range 0 to 17) and 2.00±5.00 (range 0 to 15) respectively. Wilcoxon signed-rank test indicated that the score of HAD scale not significantly different across the two domains, T=1591.0, z=1.05, p=0.292.

Comparison Mental Health Status between Diabetic Retinopathy (DR) and Glaucoma

The median scores DASS for depression and anxiety among LV patients with DR were 4.00±10.00 (range 0 to 36) and 4.00±6.00 (range 0 to 14), respectively. The median scores HAD scale for depression and anxiety among LV patients with glaucoma were 3.00±5.00 (range 0 to 17) and 3.00±5.00 (range 0 to 15) respectively. A Mann-Whitney U test indicated that all mental health score for participants with glaucoma was lower than participants with DR except anxiety score for DASS. Table 3 shows the comparison score of mental health between participants with DR and glaucoma.

Table 3: Comparison score of mental health between participants with diabetic retinopathy and glaucoma

Mental Health	Mann-Whitney Test		
	U	p	r
Depression			
DASS	782.0	0.01*	0.33
HADs	952.0	0.03*	0.21
Anxiety			
DASS	920.0	0.02*	0.23
HADs	977.0	0.05	0.19

*significance $p<0.05$

DISCUSSION

DASS and HAD Scale questionnaires used in this study indicated that the prevalence of depression among LV subjects was almost the same with the prevalence of depression among the general population in Malaysia. In Malaysia, based on a systematic review, the prevalence of depression in the general population in Malaysia was estimated to be between 8% to 12% (Ng 2014). In addition, various results reported the visual impairment and depression (Noran et al. 2009; Evans et al. 2007; Shmuely-Dulitzki et al. 1995). This is due to the vision loss can lead to depression, particularly when it stops people from pursuing valued activities (Rovner 2002). Several studies have indicated that depression associates more strongly with the functional consequences of vision loss than with its severity (Rovner et al. 1996; Mangione et al. 1999; Lenze et. al. 2001). Noran et al. (2009) also found that elderly with LV were two times more depressed compared to those with normal vision. Depression score of DASS in diabetic retinopathy and retinitis pigmentosa patients also were significantly higher than the normal subjects (Dibajnia et al. 2013). As visual impairment and depression are closely related, efficiently addressing these conditions and their consequences should be considered to be included in LV assessment.

This study also showed that LV patients not only suffered from depression but also experienced anxiety symptoms. These results are substantially higher than the prevalence of subthreshold anxiety in the general population of Malaysia (Wong et al. 2016). The results of this study confirmed the findings of previous studies that compare to the normal population, LV patients were more than likely to suffer from anxiety (Omar et al. 2014; Evans et al. 2007; Lenze et al. 2001). These findings also indicated that people with LV suffered from various severity of anxiety which was consistent with the study by Evans et al. (2007). Several other studies reported various prevalence of anxiety among visually impaired patients (9.5-30%) (van der Aa et al. 2015; Augustin et al. 2007). The variation between the

prevalence of anxiety status among visually impaired patient across studies might be due to different type of questionnaires used and the severity of visual impairment.

The highest percentage of mental health problem suffered from LV patients in this study was stress. The stress scale in DASS questionnaire measured a state of persistent arousal and tension with a small threshold for becoming saddened or frustrated (Lovibond & Lovibond 1995). Lovibond & Lovibond (1995) stated that the anxiety and stress scales overlap as there is natural continuity between the symptoms assessed by the anxiety and stress scales such as nervous tension and nervous energy. Due to this reason, it is not surprising if most of the studies assessed depression and anxiety but not stress among LV patients (Rees et al. 2009; Augustin et al. 2007; Evans et al. 2007). Omar et al. (2014) described a case report of a LV patient with diabetic retinopathy who was suffering from severe stress and severe anxiety. This showed that there was a potential overlap between anxiety and stress scales in DASS. Therefore, patients who had stress may report similar symptoms of anxiety.

There is the possibility of LV patients experience more than one mental health problems. In this study, the DASS and HAD Scale questionnaires have shown that patients with visual impairment might experience more than one mental health problems. These findings also confirmed the previous studies' result that showed LV patients suffered from depression and anxiety (Augustin et al. 2007; Evans et al. 2007). Court et al. (2014) also found that 15% of patients with visual impairment experienced two or more mental health problems. Therefore, these factors need to be taken into account as the possibility of those who experience more than one mental health problems is more difficult to undergo LV rehabilitation than those who only have one problem. In addition, different treatment and rehabilitation approaches can be used for those who experience more than one mental health problems. Harvey & Gumport (2015) stated that for each disorder, a panel of experts is formed to carefully review the scientific evidence on the best treatment/s available for each mental health problem.

Loss of visual function interferes with life's social aspects such as communication, orientation and mobility, daily activities and spirituality. All of these factors lead to a decrease in mental health. If not of these findings the examiner will never refer this patient to the clinical psychologist and be denied of further treatment. Usually, the mental health status assessment was not taken into consideration to be a part of LV assessment at LV clinic. Using DASS or HAD scale as part of the management plan for LV patients proved to be beneficial as compared to previous standard procedure. Other studies also confirmed our suspicion that the states of patient's mental health affect the rehabilitation process (van de Aa et al. 2015; Court et al. 2014; Omar et al. 2014; Tabrett & Latham 2009). This is one of the factors why LV rehabilitation fails, in that the mental health problems

were not detected and emphasized. This will lead to a low acceptance level of the LV rehabilitation process by the patient. A close relationship with other professional such as clinical psychologists is also needed for the success of such rehabilitation management. This also will encourage optometrist to work together with other professional in LV rehabilitation through a multidisciplinary approach. Thus, to confirm the status of mental health of the patient is a necessary approach during LV assessment case history taking.

CONCLUSION

Mental health has been shown to have impact on LV patients. This study showed that screening of mental health using DASS or HAD Scale questionnaires during LV assessment can contribute extra information. This highlights the needs of mental health screening in the management of LV patients. Objective information from these mental health questionnaires can be used for a better plan and management in LV rehabilitation. Furthermore, the use of DASS and HAD Scale questionnaire at this stage will further the agenda of multiple-disciplinary approach to the management of LV patients. Therefore, it is recommended that the DASS or Had Scale questionnaire be implemented during case history in LV assessment.

REFERENCES

Augustin, A., Sahel, J.A., Bandello, F., Dardennes, R., Maurel, F., Negrini, C., Hieke, K., Berdeaux, G. 2007. Anxiety and depression prevalence rates in age-related macular degeneration. *Invest Ophthalmol Vis Sci* **48**(4): 1498-503.

Bourne, R.R.A., Flaxman, S.R., Braithwaite, T., Cicinelli, M.V., Das, A., Jonas, J.B., Keeffe, J., Kempen, J.H., Leasher, J., Limburg, H., Naidoo, K., Pesudovs, K., Resnikoff, S., Silvester, A., Stevens, G.A., Tahhan, N., Wong, T.Y., Taylor, H.R., Vision Loss Expert Group. 2017. Magnitude, temporal trends, and projections of the global prevalence of blindness and distance and near vision impairment: a systematic review and meta-analysis. *Lancet Glob Heal* **5**(9): 888-97.

Breslin, F.C., Gnam, W., Franche, R-L., Mustard, C., Lin, E. 2006. Depression and activity limitations: examining gender differences in the general population. *Soc Psychiatry Psychiatr Epidemiol* **41**(8): 648-55.

Court, H., McLean, G., Guthrie, B., Mercer, S.W., Smith, D.J. 2014. Visual impairment is associated with physical and mental comorbidities in older adults: a cross-sectional study. *BMC Med* **12**(181): 1-7

Dibajnia, P., Moghadasin, M., Madahi, M.E., Keikhayfarzaneh, M.M. 2013. Depression among low vision patients. *Health Med* **27**(3): 832-6.

Evans, J.R., Fletcher, A.E., Wormald, R.P.L. 2007. Depression and anxiety in visually impaired older people. *Ophthalmology* **114**(2): 283-8.

Goldberg, D.P., Gater, R., Sartorius, N., Ustun, T.B., Piccinelli, M., Gureje, O., Rutter, C. 1997. The validity of two versions of the GHQ in the WHO study of mental illness in general health care. *Psychol Med* **27**(1): 191-7.

Harvey, A. G., Gumport, N. B. 2015. Evidence-based psychological treatments for mental disorders: Modifiable barriers to access and possible solutions. *Behav Res Ther* **68**: 1-12.

Horowitz, A., Reinhardt, J.P., Kennedy, G.J. 2005. Major and subthreshold depression among older adults seeking vision rehabilitation services. *Am J Geriatr Psychiatry* **13**(3): 180-7.

Jones, G.C., Rovner, B.W., Crews, J.E., Danielson, M.L. 2009. Effects of depressive symptoms on health behavior practices among older adults with vision loss. *Rehabil Psychol* **54**(2): 164-72.

Kempen, G.I.J.M., Ballemans, J., Ranchor, A. V., van Rens, G.H.M.B., Zijlstra, G.A.R. 2012. The impact of low vision on activities of daily living, symptoms of depression, feelings of anxiety and social support in community-living older adults seeking vision rehabilitation services. *Qual Life Res* **21**(8): 1405-11.

Lenze, E.J., Rogers, J.C., Martire, L.M., Mulsant, B.H., Rollman, B.L., Dew, M.A., Schulz, R., Reynolds, C.F. 2001. The association of late-life depression

and anxiety with physical disability: a review of the literature and prospectus for future research. *Am J Geriatr Psychiatry* **9**(2): 113-35.

Lovibond, P.F., Lovibond, S.H. 1995. The structure of negative emotional states: comparison of the Depression Anxiety Stress Scales (DASS) with the Beck Depression and Anxiety Inventories. *Behav Res Ther* **33**(3): 335-43.

Mangione, C.M., Gutierrez, P.R., Lowe, G., Orav, E.J., Seddon, J.M. 1999. Influence of age-related maculopathy on visual functioning and health-related quality of life. *Am J Ophthalmol* **128**(1): 45-53.

Ng, C.G. 2014. A review of depression research in Malaysia. *Med J Malaysia* **69**: 42-5.

Noran, N.H., Izzuna, M.G., Bulgiba, A.M., Mimiwati, Z., Ayu, S.M. 2009. Severity of visual impairment and depression among elderly Malaysians. *Asia-Pacific J Public Heal* **21**(1): 43-50.

Omar, R., Rahman, M.H.A., Knight, V.F., Mustaphal, M., Mohammed, Z. 2014. Mental health state and quality of life questionnaire in low vision assessment: a case report. *BMC Res Notes* **7**(1): 667-70.

Ramli, M., Fadzil, M.A., Zain, Z. 2007. Translation, validation and psychometric properties of Bahasa Malaysia version of the Depression Anxiety and Stress Scales (DASS). *ASEAN J Psychiatry* **8**(2): 82-9.

Ramli, M., Ramli, R., Abdullah, K., Sarkarsi, R. 2011. Concurrent validity of the depression and anxiety components in the Bahasa Malaysia version of the Depression Anxiety and Stress scales (DASS). *ASEAN J Psychiatry* **12**(1): 66-70.

Ramli, M., Rosnani, S., Fasrul, A.A. 2012. Psychometric Profile of Malaysian version of the Depressive, Anxiety and Stress Scale 42-item (DASS-42). *MJP Online Early* **21**(1): 7.

Rees, G., Fenwick, E.K., Keeffe, J.E., Mellor, D., Lamoureux, E.L. 2009. *Detection of depression in patients with low vision. Optom Vis Sci* **86**(12): 1328-36.

Rishi, P., Rishi, E., Maitray, A., Agarwal, A., Nair, S., Gopalakrishnan, S. 2017. Hospital anxiety and depression scale assessment of 100 patients before and after using low vision care: a prospective study in a tertiary eye-care setting. *Indian J Ophthalmol* **65**: 1203-8.

Rovner, B.W. 2002. Effect of depression on vision function in age-related macular degeneration. *Arch Ophthalmol* **120**(8): 1041-4.

Rovner, B.W., Zisselman, P.M., Shmuely-Dulitzki, Y. 1996. Depression and disability in older people with impaired vision: a follow-up study. *J Am Geriatr Soc* **44**(2): 181-4.

Shmuely-Dulitzki, Y., Rovner, B.W., Zisselman, P. 1995. The impact of depression on functioning in elderly patients with low vision. *Am J Geriatr Psychiatry* **3**(4): 325-9.

Stevens, G.A., White R.A., Flaxman, S.R., Price, H., Jonas, J.B., Keeffe, J., Leasher, J., Naidoo, K., Pesudovs, K., Resnikoff, S., Taylor, H., Bourne R.R.A., Vision Loss Expert Group. 2013. Global prevalence of vision impairment and blindness: magnitude and temporal trends, 1990-2010. *Ophthalmology* **120**(12): 2377-84.

Strine, T.W., Chapman, D.P., Kobau, R., Balluz, L., Mokdad, A.H. 2004. Depression, anxiety, and physical impairments and quality of life in the U.S. noninstitutionalized population. *Psychiatr Serv* **55**(12): 1408-3.

Tabrett, D.R., Latham, K. 2009. Depression and acquired visual impairment. *Optom Pract* **10**: 75-88.

van der Aa, H.P.A., Hoeben, M., Rainey, L., van Rens, G.H.M.B., Vreeken, H.L, van Nispen, R.M.A. 2015. Why visually impaired older adults often do not receive mental health services: the patient's perspective. *Qual Life Res* **24**(4): 969-78.

van der Aa, H.P.A., Van Rens, G.H.M.B., Comijs, H.C., Margrain, T.H., Gallindo-Garre, F., Twisk, J.W., van Nispen R.M. 2015. Stepped care for depression and anxiety in visually impaired older adults: multicentre randomised controlled trial. *BMJ* **h6127**: 1-9.

WHO. 2012. Global data on visual impairments 2010. Geneva: World Health Organization.

WHO. 2018. International Classification of Diseases, 11th revision (ICD-11) [World Health Organization Web site]. http://www.who.int/classifications/icd/en/ [25th October 2018].

Wong, C.H., Sultan Shah, Z.U.B., Teng, C.L., Lin, T.Q., Majeed, Z.A., Chan, C.W. 2016. A systematic review of anxiety prevalence in adults within primary care and community settings in Malaysia. *Asian J Psychiatr* **24**: 110-7.

Yahya, F., Othman, Z. 2015. Validation of the Malay version of hospital anxiety and depression scale (HADS) in hospital Universiti Sains Malaysia. *Int Med J* **22**(2): 80-2.

Yesavage, J.A., Brink, T.L., Rose, T.L., Lum, O., Huang, V., Adey, M., Leirer, V.O. 1982. Development and validation of a geriatric depression screening scale: a preliminary report. *J Psychiatr Res* **17**(1): 37-49.

Zigmond, A.S., Snaith, R.P. 1983. The hospital anxiety and depression scale. *Acta Psychiatr Scand* **67**(6): 361-70.

Received: 7 Jan 2020
Accepted: 10 Mar 2020

View publication stats
