# Supplementary material for: The psychological status of patients with delayed intravitreal injection for treatment of diabetic macular edema due to the COVID–19 pandemic
Source: PLoS One. 2023 Aug 25;18(8):e0290260. doi: 10.1371/journal.pone.0290260 (PMC10456183; doi:10.1371/journal.pone.0290260)
Supplement: Attachment — Submitted filename: Diabetic retinopathy review.pdf [file pone.0290260.s003.pdf]

Dear Editorial staff / Authors,

I have reviewed the manuscript PONE-D-22-35115, entitled “The psychological status of patients with delayed intravitreal injection for treatment of diabetic macular edema due to the COVID–19 pandemic.”; The manuscript seems well organized, but kindly you can find some points needs to be revised by the respectable authors, as below:

1- The manuscript needs to be improved for language by a native English person.
2- Depression, anxiety and other psychologic issue as COVID-19 lockdown consequences is a confounding factor; because these psychologic mood disorders could be seen in many patients regardless of having diabetic retinopathy; So reporting of these conditions by the diabetic retinopathy patients with delayed intravitreal injection, is not necessarily related to intravitreal injection postponement.
3- Just assessing *subjective* outcome measures, self-reported by the patients, is a major limiting factor in this study. Whereas other *objective* measures like best corrected visual acuity (BCVA) or macular thickness in OCT of DME could be used as outcome measures to document visual acuity changes after delayed treatment and also the severity of the diabetic retinopathy patients.
4- As mentioned in discussion, small sample size is also the other limitation factor in the study.

Best regards,
Reviewer
