# Supplementary material for: The psychological status of patients with delayed intravitreal injection for treatment of diabetic macular edema due to the COVID–19 pandemic
Source: PLoS One. 2023 Aug 25;18(8):e0290260. doi: 10.1371/journal.pone.0290260 (PMC10456183; doi:10.1371/journal.pone.0290260)
Supplement: Attachment — Submitted filename: Response to Reviewers.pdf [file pone.0290260.s004.pdf]

Below are the comments from editors and reviewers:

1. A rebuttal letter that responds to each point raised by the academic editor and reviewer(s). You should upload this letter as a separate file labeled 'Response to Reviewers'.

Thank you for your feedback. The author has written a rebuttal to answer each point made by academic editors and reviewers.

2. A marked-up copy of your manuscript that highlights changes made to the original version. You should upload this as a separate file labeled 'Revised Manuscript with Track Changes'.

Thank you for your feedback. The file will be submitted as necessary.

3. An unmarked version of your revised paper without tracked changes. You should upload this as a separate file labeled 'Manuscript'.

Thank you for your feedback. The file will be submitted as necessary.

4. If you would like to make changes to your financial disclosure, please include your updated statement in your cover letter. Guidelines for resubmitting your figure files are available below the reviewer comments at the end of this letter.

Thank you for your feedback. For your information, no financial disclosures are required for this study.

5. If applicable, we recommend that you deposit your laboratory protocols in protocols.io to enhance the reproducibility of your results. Protocols.io assigns your protocol its own identifier (DOI) so that it can be cited independently in the future. For instructions see: https://journals.plos.org/plosone/s/submission-guidelines#loc-laboratory-protocols. Additionally, PLOS ONE offers an option for publishing peer-reviewed Lab Protocol articles, which describe protocols hosted on protocols.io. Read more information on sharing protocols at https://plos.org/protocols?utm_medium=editorial-email&utm_source=authorletters&utm_campaign=protocols.

Thank you for your feedback. The lab protocols are not applicable in this study.

6. Please ensure that your manuscript meets PLOS ONE's style requirements, including those for file naming. The PLOS ONE style templates can be found at https://journals.plos.org/plosone/s/file?id=wjVg/PLOSOne_formatting_sample_main_body.pdf and https://journals.plos.org/plosone/s/file?id=ba62/PLOSOne_formatting_sample_title_authors_affiliations.pdf

Thank you for your feedback. The manuscript has been changed to follow the style template as required

7. In your Data Availability statement, you have not specified where the minimal data set underlying the results described in your manuscript can be found. PLOS defines a study's minimal data set as the underlying data used to reach the conclusions drawn in the manuscript and any additional data required to replicate the reported study findings in their entirety. All PLOS journals require that the minimal data set be made fully available. For more information about our data policy, please see http://journals.plos.org/plosone/s/data-availability.

Thank you for your feedback. The minimum sample size required for this study has been included in the methodology. Based on the sample size calculation for a finite population, the required sample size was 120 subjects. However, missing data and dropouts occurred during the study period, resulting in a final sample size of 86 subjects. While this is less than the original calculated sample size, we made efforts to handle the missing data appropriately and ensure the integrity of the analysis.

In the limitations section of our study, we have acknowledged the final sample size of 86 subjects and discussed the potential impact on the statistical power and generalizability of our results. We appreciate your feedback, and recognize the importance of sample size considerations in research studies. Your input will be considered in future studies to ensure that appropriate sample sizes are achieved and maintained.

8. Upon re-submitting your revised manuscript, please upload your study's minimal underlying data set as either Supporting Information files or to a stable, public repository and include the relevant URLs, DOIs, or accession numbers within your revised cover letter. For a list of acceptable repositories, please see http://journals.plos.org/plosone/s/data-availability#loc-recommended-repositories. Any potentially identifying patient information must be fully anonymized.

Thank you for your feedback. Supporting information file will be provided during revised manuscript submission.

9. PLOS requires an ORCID iD for the corresponding author in Editorial Manager on papers submitted after December 6th, 2016. Please ensure that you have an ORCID iD and that it is validated in Editorial Manager. To do this, go to 'Update my Information' (in the upper left-hand corner of the main menu), and click on the Fetch/Validate link next to the ORCID field. This will take you to the ORCID site and allow you to create a new iD or authenticate a pre-existing iD in Editorial Manager. Please see the following video for instructions on linking an ORCID iD to your Editorial Manager account:

https://www.youtube.com/watch?v=_xcclfuvtxQ.

Thank you for your feedback. The ORCID ID has been linked to the author's PLOS account and has been updated in the author profile.

10. Please ensure that you include a title page within your main document. You should list all authors and all affiliations as per our author instructions and clearly indicate the corresponding author.

Thank you for your feedback. The changes have been made as required.

11. Please amend your manuscript to include your abstract after the title page.

Thank you for your feedback. Abstract of the study has been added in manuscript as required.

12. We note you have included a table to which you do not refer in the text of your manuscript. Please ensure that you refer to Table 2 and 4 in your text; if accepted, production will need this reference to link the reader to the Table.

Thank you for your feedback. The Table 2 and 4 have been mentioned in the text accordingly.

13. Please work on introductory lines on Abstract (as well as work on the methods and discussion subsections)

Thank you for your feedback. The research abstract has been added into the manuscript. Improvement also has been made on the Methodology and Discussion sections.

14. The Introduction is somewhat confusing while changing from a topic to another - maybe it'd be better detail what has been found in previous studies (in terms of structures bus also processing) and how to overcome the limitations;

Thank you for your feedback. Improvement on the introduction has been made as suggested.

*15. The authors *really* need to work on eligibility criteria (more information on background is needed: patients were excluded if had substance abuse (references), any mental disease (references), any neurological disease (references) etc. The same is worrying for the OCT. Several studies have found defects on functioning, but these are solely because of diabetic oedema or any macular oedema: briefly, this bias results because the OCT and anomaloscope (in some cases) were not properly used.*

Thank you for your feedback. The eligibility criteria (inclusion and exclusion criteria) have been added to the methodology. The exclusion criteria were also mentioned as suggested in the comment. Regarding the OCT, this device is a gold standard to diagnosed diabetic macular edema with central involvement and for monitoring response to treatment. In our experience, we did not find any malfunction during the test and the device was used properly. It is a standardized tool for the diagnosis of DME in our patients.

*16. The authors *really* need to use Bayesian statistics due to the sample size (effects, posterior odds, the Bayes Factors etc*

Thank you for your feedback. We appreciate your suggestion to use Bayesian statistics in our study due to the sample size. However, after careful consideration, we have decided to use the frequentist approach. This choice is consistent with our study design and objectives, adheres to common practices in our field, allows for easy interpretation of results, and provides reliability. While we acknowledge the merits of Bayesian statistics, we believe that the traditional frequentist approach was appropriate for our study desig. We thank you for your input and we hope that this explanation clarifies our rationale.

17. Also, the stats section needs to be refined

We appreciate your feedback. In our study, we conducted a non-parametric analysis considering the distribution of our data. To assess the normality of the data, we used Shapiro-Wilk analysis. The relationship between the DASS score and the other variables was evaluated using appropriate non-parametric tests such as Kendall rank correlation, Mann-Whitney U test, and Kruskal-Wallis test. To further group comparisons, a post hoc test with Bonferroni adjustment was performed.

We believe that this type of analysis is suitable for our data set, given the nonparametric nature and distributional characteristics of our variables. We have considered feedback from another reviewer and made sure to include additional details in the Results section to improve the clarity

of our findings. We hope that this clarification demonstrates the appropriateness of the statistical analysis methods we chose for our study and addresses the reviewer's concerns.

18. Based on these concerns, is somewhat hard to follow Discussion

Thank you for your feedback. Improvement on the Discussion section has been made as suggested.

JABATAN OFTALMOLOGI
Tingkat 9, Blok Klinikal Hospital Canselor Tuanku Muhriz, HCTM
Jalan Yaacob Latif, Bandar Tun Razak, 56000 Cheras, Kuala Lumpur,
Malaysia.
Telefon:**+603-91455981/5982/5998** Faksimili:**+603-91456673**

Reviewer Comments to the Author

Reviewer 1:

Dear Editorial staff / Authors,

I have reviewed the manuscript PONE-D-22-35115, entitled "The psychological status of patients with delayed intravitreal injection for treatment of diabetic macular edema due to the COVID–19 pandemic."; The manuscript seems well organized, but kindly you can find some points needs to be revised by the respectable authors, as below:

1. The manuscript needs to be improved for language by a native English person.

Thank you for your feedback. Improvement in the writing has been made as suggested.

2. Depression, anxiety and other psychologic issue as COVID-19 lockdown consequences is a confounding factor; because these psychologic mood disorders could be seen in many patients regardless of having diabetic retinopathy; So reporting of these conditions by the diabetic retinopathy patients with delayed intravitreal injection, is not necessarily related to intravitreal injection postponement.

Thank you for your feedback. We agree that depression, anxiety, and other mental health problems are consequences of COVID -19 lockdown. In our study, we assessed the other possible confounding factors of patients, including demographics, comorbidities other than diabetes, and life aspects that they felt were affected by the pandemic. From our study, certain demographic data, namely race, marital status, and education level, showed a significant association with the DASS score in these groups. There was no association between the other medical comorbidities and the DASS score among the subjects. However, as mentioned by the reviewer, further studies are needed to find confounding factors other than delay of intravitreal injection and demographic factors as mentioned above.

3. Just assessing subjective outcome measures, self-reported by the patients, is a major limiting factor in this study. Whereas other objective measures like best corrected visual acuity (BCVA) or macular thickness in OCT of DME could be used as outcome measures to document visual acuity changes after delayed treatment and also the severity of the diabetic retinopathy patients.

Thank you for your feedback. We appreciate your suggestion to include BCVA, macular thickness in OCT, and severity of diabetic retinopathy to correlate with degree of visual loss and psychological impact. However, in this study, we only assessed subjective visual acuity and not objective visual acuity. We apologize that evaluation of visual function based on BCVA, OCT macula, and severity of diabetic retinopathy is not part of the aim of this study.

4. As mentioned in discussion, small sample size is also the other limitation factor in the study.

Thank you for your feedback. We acknowledge the limitation of the small sample size in our study. Despite this limitation, we carefully designed our study and performed appropriate statistical analyses. Given the limitations and availability of participants in our study setting, it was challenging to achieve a larger sample size. However, we believe that our study still contributes valuable insights related to our research question. In the Discussion section, we specifically noted this limitation and its potential impact on the interpretation of our results. We emphasize the need of future studies with larger sample sizes to validate and extend our findings. Your feedback is valuable, and we appreciate your acknowledgment of this limitation in our study.

Best regards,
Reviewer

Reviewer 2:

Comment: In the present study authors have investigated delayed treatment of DME due to the COVID-19 pandemic causes depression, anxiety, and stress in a small proportion of patients. The paper is interesting; however, as the author said, the limitation of this study is the small size of the subjects, besides, there are great differences between different races. I have two comments which need to be addressed.

1. In table 1, the unemployment rate is 90.7% in this project? As the author said, the government of Malaysia has implemented a movement control order for a total of 32 months. In my opinion, such high unemployment rate and movement control may lead to depression, anxiety, and stress also.

Thank you for your feedback. As mentioned in the manuscript, the unemployment rate was relatively high during the data collection. However, this unemployment rate was not directly attributable to unemployment due to the pandemic COVID -19. The unemployment rate also includes elderly patients who are currently unemployed after retirement or for other reasons that may predate the pandemic. Analysis of the relationship between the unemployment rate and the DASS score was performed, and we found no significant relationship between the unemployment rate and the DASS score.

2. Why did you choose respondents with a median age of 69 years old？

Thank you for the question. As mentioned in the manuscript, the median age of the subjects was 69 years old. For your information, the age of the recruited subjects is in accordance with the inclusion criteria. Since the median age of our subjects is 69 years, there is no particular reason to recruit subjects within this median age.

JABATAN OFTALMOLOGI
Tingkat 9, Blok Klinikal Hospital Canselor Tuanku Muhriz, HCTM
Jalan Yaacob Latif, Bandar Tun Razak, 56000 Cheras, Kuala Lumpur,
Malaysia.
Telefon:**+603-91455981/5982/5998** Faksimili:**+603-91456673**

Reviewer 3:

The aim of this study is to assess the psychological status of DME patients with delayed anti-VEGF treatment during the pandemic. The key points of this study are the definition of "delayed anti-VEGF treatment" and how "psychological status" was measured.

Overall impression: Well-structured and very well written.

1. Introduction is well written. However, I do not think the reader has a concrete picture of the impact of PANDEMIC on MENTAL HEALTH. Although You mention it as "Psychological implications during outbreaks of transmittable diseases such as severe acute respiratory syndrome (SARS) and equine influenza have also previously been reported (4)", can you even mention more specific?

Thank you for your feedback. Impacts of pandemic on mental health have been reported from several articles, and have been included in the Introduction section.

2. DASS-21 may seem to be a suitable METHOD for this study. However, ophthalmology-related professionals are often unaware of the extent to which DASS-21 adequately reflects this patient-centered outcomes. So, please mention in detail the validity of the DASS-21 for ascertaining mental status, based on previous reports (validation, reliability,..).

Thank you for your feedback. DASS-21 is used in many articles to assess mental health and psychological wellbeing. Good reliability and validity of the DASS has also been demonstrated in previous studies. The validity of the DASS in previous reports and examples have been included in the Method section.

3. A definition of "delay" is one of the key points in this study. You mentioned only "Patients who had delayed at least once (one month) of intravitreal anti-VEGF treatment for DME aged between 20 to 80 years were included in this study." Almost no problem, but what do you define as "delay"? For example, if you are administering intravitreal anti-VEGF treatment as fixed or TAE, it may be easier to define "delay" since it is calculated from the scheduled injection date. However, when the PRN is administered, whether or not the injection is given on the same day is based on the OCT results, so it is questionable to define "delay" as starting from the date of the return visit.

JABATAN OFTALMOLOGI
Tingkat 9, Blok Klinikal Hospital Canselor Tuanku Muhriz, HCTM
Jalan Yaacob Latif, Bandar Tun Razak, 56000 Cheras, Kuala Lumpur,
Malaysia.
Telefon:**+603-91455981/5982/5998** Faksimili:**+603-91456673**

Thank you for your feedback. The subjects recruited were those who required monthly and scheduled treatment. Delay is defined as the postponement of treatment from the last scheduled intravitreal injection. Those who received a regular monthly injection and TAE were included in the study. PRN regime was not included as a study subject.

4. In the result section, I think adding the mean, median, range, and histogram for the DELAY interval would improve the external validity of the study results.

Thank you for your feedback. The mean, median, range and histogram for the delay interval have been added in the Result section as suggested.

5. With respect to INCOME, how about including the US dollar equivalent in the table?

Thank you for your feedback. The income in US dollar equivalent has been added in the table accordingly.

6. Regarding the result of Table 7, the DASS-21 scores of the group more than 12w ago had the lowest scores. Why is the dose-response relationship not between the DELAY period and the DASS score? It needs to be clearly mentioned in the discussion.

Thank you for your feedback. Table 7 shows the comparison of the DASS scores with the last intravitreal injections received by the patients. For your information, the last intravitreal injection the patients received was the treatment delay time. Therefore, this table represents the relationship between the delay times and the DASS score. As shown in the table, those who experienced delay of more than 12 weeks had the lowest mean rank DASS score. The Discussion section has been improved as suggested.

7. Regarding the post hoc test, I assume that you are correcting for comparisons among all groups. You need to mention the detailed results.

Thank you for your feedback. Yes, the post-hoc test was performed to compare all groups with a significant p-value in the non-parametric test. The detailed results of the pairwise comparison in the post-hoc test have been added in the tables.

JABATAN OFTALMOLOGI
Tingkat 9, Blok Klinikal Hospital Canselor Tuanku Muhriz, HCTM
Jalan Yaacob Latif, Bandar Tun Razak, 56000 Cheras, Kuala Lumpur,
Malaysia.
Telefon:**+603-91455981/5982/5998** Faksimili:**+603-91456673**

8. Are there any differences in the delay period between genders or social backgrounds? Sex differences in time to treatment have been reported for retinal detachment,1 and there are significant social implications for sex differences in the impact of COVID-19.

Thank you for your feedback. Regarding this question, our data showed no differences in the delay period among genders. However, there were significant difference of DASS score between races, educational level, and marital status as shown in the Result section.

9. What was the background for the racial differences in THE SCORE this time? Please mentioned in the discussion section. Why the Chinese people had the highest scores.

Thank you for your feedback. The mean rank DASS score for depression and stress is highest among Chinese, followed by Indians and Malays. We tried to find out why the Chinese subjects had a higher score, but we could not find any other factors/causes that contributed to this outcome. Discussion on this was added as suggested.

10. Please mentioned the detail of the method for analyzing the relationship between socio-demographic factors and DASS-21 score. Only P values are reported in Table 11, which is not understandable, especially for continuous variables.

Thank you for your feedback. In this regard, we have added the details of the method in the table for each factor in the table accordingly.

JABATAN OFTALMOLOGI
Tingkat 9, Blok Klinikal Hospital Canselor Tuanku Muhriz, HCTM
Jalan Yaacob Latif, Bandar Tun Razak, 56000 Cheras, Kuala Lumpur, Malaysia.
Telefon:**+603-91455981/5982/5998** Faksimili:**+603-91456673**
