# Supplementary material for: The psychological status of patients with delayed intravitreal injection for treatment of diabetic macular edema due to the COVID–19 pandemic
Source: PLoS One. 2023 Aug 25;18(8):e0290260. doi: 10.1371/journal.pone.0290260 (PMC10456183; doi:10.1371/journal.pone.0290260)
Supplement: Attachment — Submitted filename: Re-review.pdf [file pone.0290260.s005.pdf]

I really appreciate the authors' point to point response to the comments; But, in spite of having a very well written manuscript, some of the respectable author's replies to the comments seem not convincing. Below you can find some major concerns, regarding this issue:

- ***Data analysis:*** mostly using the frequentist approach instead of the Bayesian statistics for data statistical analysis.
- ***Methods:*** not using the OCT for DME diagnosis and intra-vitreal anti-VEGF injection.
- ***Methods:*** Being prevalent of neurotic disorders like depression and anxiety during COVID-19 pandemic due to multifactorial aspect is a major confounding factor, as it can not be considered just as the consequence of delayed treatment of diabetic retinopathy.
- ***Methods:*** non defined the exact definition of delay time for anti-VEGF intravitreal injection. As already known, basically the making decision for anti VEGF intravitreal injection in clinic is based on the fundoscopy examination and OCT; and not just the already scheduled time for injection.
- ***Outcome measures:*** just using the patients' subjective outcome measures instead of the objective ones like BCVA and OCT, which strongly influence the accuracy and validity of the study.
- ***Conclusion:*** As a matter of fact, each longstanding or life-threatening illness such as diabetes mellitus, coronary heart disease, cancer or delayed diabetic macular edema treatment can cause depression or anxiety. As a whole, what is the added message of the article to this already known principle?

  Best regards,
