# Supplementary material for: The psychological status of patients with delayed intravitreal injection for treatment of diabetic macular edema due to the COVID–19 pandemic
Source: PLoS One. 2023 Aug 25;18(8):e0290260. doi: 10.1371/journal.pone.0290260 (PMC10456183; doi:10.1371/journal.pone.0290260)
Supplement: Attachment — Submitted filename: Response to reviewer.docx [file pone.0290260.s006.docx]

Below are the comments from editors and reviewers:

Editor comments

Thank you for your valuable submission. I call out authors to consider and address the remaining concerns of Reviewer 1.

Actually, there are two concerns that are really important:

1. What is new, beyond we already know?

*Thank you for your feedback. Firstly, our study specifically focuses on the relationship between delayed treatment for diabetic macula edema (DME) and its association with depression, anxiety and stress. While we recognized that delayed treatment can lead to psychological distress in general, our study specifically evaluates this psychological wellbeing within the context of DME patients.*

*Secondly, by incorporating self-reported experience and subjective outcome measures, we provide a comprehensive view of the psychological impact of delayed intravitreal anti-VEGF treatment among DME patients. We also highlight the importance of prompt intervention and timely treatment for DME not only for vision-related outcomes but also for mental well-being. By emphasizing the significant association between delayed treatment and increased risk of depression, anxiety and stress, we point up the need for comprehensive care that addresses both the physical and psychological aspects of this condition.*

*In summary, our study contributes new insights by focusing on subjective outcome measures and emphasizing the importance of timely intervention. These findings provide a more comprehensive understanding of the psychological impact of delayed treatment for DME patients particularly during pandemic.*

2. Also, please consider Bayesian as I also agree with the reviewer's point on the need to use robust analyses.

*Thank you for your feedback. A Bayesian analysis using Stata version 14 utilizing the Metropolis-Hastings algorithm was performed to estimate the mean and median of the DAS score to the delayed injection and impact of Covid-19. The analysis is shown in Table 7.*

Reviewers comments

Reviewer 1:

I really appreciate the authors’ point to point response to the comments; But, in spite of having a very well written manuscript, some of the respectable author’s replies to the comments seem not convincing. Below you can find some major concerns, regarding this issue:

1. Data analysis: mostly using the frequentist approach instead of the Bayesian statistics for data statistical analysis.

*Thank you for your feedback. Additional data analysis using Bayesian statistic is shown in Table 7.*

2.Methods: not using the OCT for DME diagnosis and intra-vitreal anti-VEGF injection.

*Thank you for your feedback. In our clinical practice, we routinely use OCT as a diagnostic tool for diabetic macular edema (DME) and as a mean to decide the initiation of intravitreal anti-VEGF injections, as well as to monitor the progression of the disease. This statement has been in included in the Methodology section.*

3. Methods: Being prevalent of neurotic disorders like depression and anxiety during COVID-19 pandemic due to multifactorial aspect is a major confounding factor, as it cannot be considered just as the consequence of delayed treatment of diabetic retinopathy.

*Thank you for your feedback. When subjects were recruited, exclusion criteria included those who had a psychiatric history or mental illness, including depression and anxiety. The prevalence of depression and anxiety during the COVID -19 pandemic due to many aspects is inevitable. In our opinion, to study the other factors that may contribute to depression and*

*anxiety is another objective that can be carried out. In this study, we assessed the association between the DAS scores and delayed treatment for anti-VEGF. As stated in the result, there was a significant DAS score among those who vision was affected due to the delayed treatment. The impacts of COVID-19 and life aspects that affected due to COVID-19 did not show significant DAS scores among our subjects.*

4. Methods: non defined the exact definition of delay time for anti-VEGF intravitreal injection. As already known, basically the making decision for anti VEGF intravitreal injection in clinic is based on the fundoscopy examination and OCT; and not just the already scheduled time for injection.

*Thank you for your feedback. In our study,* *delay time for anti-VEGF defined as those who had been delayed from a scheduled injection. In our clinical practice, decision of injection for DME are those who has evident of DME through funduscopy examination and OCT finding. Each clinic visit, patients will need to do visual acuity assessment, fundus examination and OCT macula to monitor disease progression. After antiVEGF injection, patients will be given a scheduled appointment for next antiVEGF injection during each clinic visit. Therefore, those who experienced delay of injection are those who had delayed treatment from scheduled appointment for injection.*

5. Outcome measures: just using the patients’ subjective outcome measures instead of the objective ones like BCVA and OCT, which strongly influence the accuracy and validity of the study.

*Thank you for your feedback. We understand your concern about relying solely on subjective measures from patients rather than objective measures such as BCVA and OCT. Objective measures are indeed important in ensuring the accuracy and validity of a study. However, in this study, our focus was on incorporating the patients' perspectives and experiences, which are valuable in understanding the impact of delayed treatment on their mental well-being. In our opinion, subjective measures will gain valuable insights into the patients' perspectives, which can provide a more comprehensive understanding of the impact of delayed treatment for anti-VEGF and its association with DAS. We believe that subjective measures contribute to a more holistic evaluation of the patients' experiences, more than what can be acquired through objective measures alone.*

*We acknowledge that objective measures are vital in many research contexts, and they have their own strengths in assessing clinical outcomes. However, for the specific objectives of our*

*study, we believe that the subjective outcome measures provide us a deeper understanding of the mental health implications for patients affected by the delayed eye injection.*

6. Conclusion: As a matter of fact, each longstanding or life-threatening illness such as diabetes mellitus, coronary heart disease, cancer or delayed diabetic macular edema treatment can cause depression or anxiety. As a whole, what is the added message of the article to this already known principle?

*Thank you for your feedback. The added message of our study lies in the specific context of delayed treatment for diabetic macular edema and its impact on mental health. By focusing on this condition, we aim to provide insight on the psychological consequences of treatment delays and their potential contribution to depression, anxiety and stress in affected patients.*

*Our study shows a significant association between delayed anti-VEGF treatment for DME and levels of depression, anxiety, and stress. By examining the relationship between delayed treatment and subjective outcome measures, we hope to contribute to a deeper understanding of the psychosocial aspects surrounding this condition. Apart from that, this study also emphasizes the importance of timely treatment for DME, not only for vision-related outcomes but also for the mental well-being of patients. By highlighting the significant association between delayed treatment and increased risk of depression, anxiety and stress, we hope to raise awareness among ophthalmologist and clinicians about the need for prompt intervention in managing this eye condition.*
